# A randomized, open-label two-period crossover pilot study to evaluate the relative bioavailability in the fed state of atovaquone-proguanil (Atoguanil™) versus atovaquone-proguanil hydrochloride (Malarone®) in healthy adult participants

**DOI:** 10.1007/s00210-024-03245-x

**Published:** 2024-06-25

**Authors:** Andrea Kuemmerle, Denis Gossen, Michael W. Marx, Ulrike Lorch, Maja Szramowska, Ashok Kumar, Dharmendra Singh, Satinder Singh, Hanu Ramachandruni, Byju Thankachen, Swapnil Kore, Myriam El Gaaloul, Isabelle Borghini-Fuhrer, Stephan Chalon

**Affiliations:** 1 Medicines for Malaria Venture, ICC – Block G, 3rd floor, 20, Route de Pré-Bois, PO Box 1826, 1215 Geneva, Switzerland; 2 Mangareva SRL, Kraainem, Belgium; 3 ICON Clinical Research Germany GmbH, Langen, Germany; 4 Richmond Pharmacology Ltd, London, UK; 5 PharmaKinetic Ltd, Quorn, UK; 6 IPCA Laboratories Limited, Mumbai, India

**Keywords:** Atovaquone-proguanil, Bioavailability, Pharmacokinetics, Malaria

## Abstract

Atoguanil™ is a novel complex of atovaquone (ATV) and proguanil (PG) with enhanced ATV bioavailability compared to Malarone®. This pilot study assessed whether the relative bioavailability (F_rel_) of ATV, PG, and the primary PG metabolite cycloguanil (CG) following a single oral dose in the fed state of *Atoguanil* was similar to *Malarone* despite a 50% lower ATV dose. This open-label, single-dose, randomized 2-period, 2-treatment, balanced crossover study was conducted between 17th November 2021 and 18th March 2022. Eligible participants (aged 18–55 years) were randomized (1:1) in period 1 to *Atoguanil* (ATV/PG 500/348 mg) or *Malarone* (ATV/PG hydrochloride 1000/400 mg) administered following a high-fat, high caloric meal. After a 24-day washout period, participants crossed treatment arms. For the doses tested, F_rel_ was assumed similar if 90%CIs were between 80 and 125% for the geometric mean ratio of the least square mean differences for each exposure parameter. In 15 evaluable participants, F_rel_ was similar for ATV C_max_ (93.6% [90%CI 83.6, 104.9]) but not AUC_0-inf_ (77.8% [67.4, 89.8]), for PG AUC_0-inf_ (95.6% [92.1, 99.2]) but not C_max_ (82.4% [75.8, 89.5]), and for both CG C_max_ (100.8% [95.0, 107.0]) and AUC_0-inf_ (102.9% [98.4, 107.7]). Nine adverse events occurred; all were of mild severity and not considered treatment related. At the doses tested, ATV F_rel_ was lower following *Atoguanil* versus *Malarone* based on AUC_0-inf_, though when adjusted for dose F_rel_ increased by 156%. Both drugs were well tolerated with no safety concerns. ClinicalTrials.gov: NCT04866602 (April 26th, 2021)

## Introduction

Most deaths from malaria occur in African children under 5 years of age with *Plasmodium falciparum* infection (World Health Organization 2023). Thus, preventing *P. falciparum* malaria in children is a key intervention in reducing the risk of mortality and morbidity associated with severe malaria.

The World Health Organization recommends seasonal malaria chemoprevention (SMC) for children aged 3 months to 5 years in areas with highly seasonal malaria transmission (World Health Organization 2022). A monthly dose of sulfadoxine-pyrimethamine + amodiaquine (SPAQ) is administered for 3 to 4 consecutive months to reduce malaria cases, hospitalizations, and deaths (Adjei et al. 2022). SMC deployment is currently limited to the sub-Sahel region due to a high prevalence of SPAQ-resistant *P. falciparum* in southern, central, and eastern Africa (Amimo et al. 2020). Moreover, the increased prevalence of molecular markers of drug resistance in areas where SPAQ is currently deployed for SMC is concerning (Ndiaye et al. 2023).

The antimalarial drug Malarone® is a fixed-dose combination of atovaquone (ATV) and proguanil (PG), formulated as the hydrochloride salt (PG_HCl_). The combination is active against *P. falciparum* liver and blood stages, being registered for *P. falciparum* malaria prophylaxis and the treatment of uncomplicated malaria or uncomplicated *P. falciparum* malaria (Croft 2010; Nakato et al. 2007; Blanshard and Hine 2021). *Malarone* has a favorable safety profile and good tolerability (Nakato et al. 2007; Blanshard and Hine 2021; Bustos et al. 1999; Overbosch 2003). Despite the patent for *Malarone* expiring in 2013, generic ATV-PG_HCl_ has not been considered for use in chemoprevention in malaria endemic regions because of its high cost.

The hydroxynaphthoquinone ATV is a competitive inhibitor of ubiquinol, disrupting the parasite mitochondrial electron transport chain at the bc1 complex. Elimination is mainly via the liver through bile clearance (Zsila and Fitos 2010), with no evidence of metabolization and an elimination half-life of ~50–84 h (Rolan et al. 1997; Nixon et al. 2013). ATV is highly protein bound (> 99.5%) and lipophilic (log P 5.1) with poor oral bioavailability (Nixon et al. 2013). Administration with a high-fat meal increases the area under the concentration–time curve (AUC) by 3.3-fold and maximum concentration (C_max_) by 5.3-fold (Rolan et al. 1994). However, even when taken with food, as per the *Malarone* label recommendation, ATV maximum bioavailability was 23% (Hussein et al. 1996). Because of the low exposures achieved, ATV monotherapy has limited efficacy and parasite resistance readily emerges (Looareesuwan et al. 1996). However, antimalarial efficacy is enhanced synergistically when ATV is combined with PG, reducing the risk of recrudescence and ATV resistance emergence (Looareesuwan et al. 1996; Lin et al. 2021). Nevertheless, the high ATV dose and a relatively expensive manufacturing process results in a high cost-of-goods for *Malarone*, limiting its use for malaria prevention in endemic countries.

Proguanil is a biguanide derivative that is converted to 4-chlorophenyl biguanide and the active metabolite cycloguanil (CG), a parasite dihydrofolate reductase (DHFR) inhibitor. Metabolism is via cytochrome P450 (CYP) 2C19 and CYP3A4 (Pudney et al. 1999). Genetic polymorphism in CYP2C19 results in higher PG and lower CG plasma concentrations in poor metabolizers (Beerahee 1999). The synergy between ATV and PG does not appear to result from DHFR inhibition. Rather, PG enhances the ATV provoked collapse in mitochondrial membrane potential without affecting electron transport inhibition (Srivastava and Vaidya 1999). This explains why ATV-PG_HCl_ does not require dose adjustment in populations with a high prevalence of poor PG metabolizers and retains efficacy against PG-resistant parasites (Hussein et al. 1996; Srivastava and Vaidya 1999).

To enhance ATV bioavailability and potentially reduce the required dose, a novel complex of ATV with PG free base has been developed (Atoguanil™). *Atoguanil* dissociates into ATV and PG in vivo when it comes into contact with gastro-intestinal fluids. The resulting ATV is found to be more polar and more soluble in aqueous media compared to free ATV from *Malarone*, resulting in better absorption and bioavailability *in vivo*. ATV from *Atoguanil* showed a more than three-fold higher dissolution (over 90%) compared to ATV from *Malarone*. In a preclinical pharmacokinetic study in the rabbit, this difference in dissolution translated into an approximately twofold increase in ATV oral bioavailability with *Atoguanil* compared to *Malarone*, and similar findings were reported in rats (Medicines for Malaria Venture, data on file).

This pilot study in healthy participants compared the pharmacokinetic (PK) profiles and drug exposures for ATV, PG, and CG obtained after *Atoguanil* or *Malarone* administration, containing an ATV dose of 500 mg or 1000 mg, respectively. The aim was to determine whether a twofold reduction in the ATV dose with *Atoguanil* was feasible while maintaining appropriate therapeutic exposure. Such an improved bioavailability would potentially lead to a significant cost reduction with *Atoguanil* versus *Malarone*, increasing affordability for use in malaria chemoprevention, including SMC, in endemic regions.

## Methods

### Study design

This open-label, single-dose, randomized 2-period, 2-treatment balanced crossover pilot study evaluated the PK profile and relative bioavailability (F_rel_) of single-dose *Atoguanil* tablets in healthy adults relative to *Malarone* in the fed state.

Study drugs were *Atoguanil* (Ipca Laboratories Ltd, Mumbai, India), comprising ATV 500 mg plus PG free base 348 mg administered as four tablets of 125:87 mg; and *Malarone* (GSK, Ware, UK) comprising ATV 1000 mg plus PG_HCl_ 400 mg (equivalent to PG 348 mg free base) administered as four tablets of 250:100 mg. The *Malarone* dose administered was the approved dose for treating acute uncomplicated malaria as chemoprevention currently requires a full course of antimalarial treatment (World Health Organization 2022). The *Atoguanil* ATV dose was reduced from 1000 to 500 mg, anticipating similar ATV exposures after oral administration of *Atoguanil* and *Malarone*. Exposure to PG from *Atoguanil* was predicted to be similar to that from *Malarone*. Thus, PG dose (free base) in *Atoguanil* matched the PG dose (hydrochloride) in *Malarone*.

As *Malarone* is administered with food or a milky drink, the fed condition was selected to assess bioavailability for both formulations. The study was conducted at Richmond Pharmacology Ltd (London, UK) between 17th November 2021 and 18th March 2022 (ClinicalTrials.gov: NCT04866602 registered 26th April, 2021; EudraCT number 2021-003422-69).

The primary objective was to determine the F_rel_ of ATV, PG, and CG derived from a single oral dose of *Atoguanil* compared to a single oral dose of *Malarone* in the fed state at the doses administered. Secondary objectives were to further describe the single-dose PK properties of ATV, PG, and CG in healthy participants following a single dose of *Atoguanil* or *Malarone* in the fed state, to assess the safety and tolerability of *Atoguanil*, and further document the safety/tolerability of *Malarone*.

The study conformed to Good Clinical Practice as per the International Council for Harmonization of Technical Requirements for Pharmaceuticals for Human Use Good Clinical Practice Guidelines, the Declaration of Helsinki, and all applicable local laws and guidance. All participants provided informed signed consent before undertaking any study procedure. The study protocol including one amendment were reviewed and approved by the London Bridge Research Ethics Committee.

### Participants

Eligible participants were male and female, including those of child-bearing potential, aged between 18 and 55 years, with bodyweight 50 to 80 kg and a body mass index 18 to 25 kg/m^2^. Participants were in good health, non-smokers, with no significant medical history, or clinically relevant abnormalities at screening based on physical examination, vital signs, clinical laboratory evaluations, or electrocardiogram (ECG). All participants had to use highly effective contraception, unless post-menopausal or sterilized. Pregnant and lactating women or partners of pregnant or lactating women were excluded. Key exclusion criteria were known hypersensitivity to ATV or PG, any significant underlying disease, condition or infection, including HIV, hepatitis B and drug and food allergies, current or history of psychiatric illness, any condition affecting drug absorption, a history of photosensitivity, a history or clinical evidence of substance or alcohol abuse, receipt of an investigational drug within 90 days or 5 half-lives, any moderate/strong inhibitor/inducer of CYP450 within 30 days or 5 half-lives, or any other drug in the previous 7 days or 10 half-lives or herbal supplement within 30 days, or any other significant disease or disorder considered to put the participant at risk.

### Procedures

Participants were screened within 30 days of trial start (Day −1). In period 1, participants were randomized (1:1) to *Atoguanil* or *Malarone* according to a computer-generated randomization schedule with cross-over to the other treatment in period 2. For each study period, study medication was administered on Day 1, participants discharged on Day 4, and outpatient follow-up visits conducted on Days 8, 15, and 22. There was a minimum 24-day washout period between each study period. Post-screening procedures are shown in Table 1.

**Table 1:** Post-screening assessments

Assessment	Day							
	−1	1	2	3	4	8	15	22
Medical history/concomitant medication	●							
Drug and alcohol tests	●							
Pregnancy test	●							
Inclusion/exclusion criteria	●	●						
Physical examination		●	●	●				●
Vital signs	●	●	●	●	●			●
Adverse events^a^	●	●	●	●	●	●	●	●
Clinical laboratory tests^b^	●	●	●	●		●		●
12-lead ECG	●	●	●	●	●			●
PK blood sampling^c^		●	●	●	●	●	●	●

^a^Adverse events were monitored continuously and spontaneous serious adverse events were reported up until 30 days after the final study visit on Day 22

^b^Biochemistry, hematology, coagulation, urinalysis

^c^Samples were collected relative to dosing at −1, −0.5, 0, 1, 2, 2.5 3, 3.5, 4, 4.5, 5, 6, 8, 10, 12, 16, 24, 48, and 72 h for ATV, PG, and CG and at 168, 336, and 504 h for ATV only

Before treatment administration, participants fasted overnight for at least 10 h, then consumed an FDA-type high-fat/calorie breakfast (796 kcal, 45.2 g/406.8 kcal of fat). Study drug was administered at completion of the breakfast being consumed entirely within 30 min of serving and with 240 mL of water. No additional food was allowed for 4 h post-dose, and additional water intake was not allowed 1 h before and after dosing. Participants were asked to not lie fully recumbent for 2 h post-dosing. Blood samples were collected for determination of ATV, PG, and CG plasma concentrations using a validated method. The lower limit of quantification (LLOQ) was 100 ng/mL for ATV, 5.00 ng/mL for PG and 1.50 ng/mL for CG.

### Endpoints

The primary endpoints were F_rel_ for ATV at the doses tested for C_max_, the AUC from time zero to last detectable plasma concentration (AUC_0-t_), AUC from time zero to 72 h (AUC_0-72h_), AUC from time zero to 168 h (AUC_0-168h_), and AUC from time zero to infinity (AUC_0-inf_). Also, the F_rel_ for PG and CG for C_max_ and AUC_0-inf_ at the doses tested were primary endpoints. Although AUC_0-inf_ is recommended as the most relevant measure for assessing F_rel_ (United States Food and Drug Administration 2022), as ATV has a long terminal half-life (t_1/2_), additional AUC endpoints were considered in case AUC_0-inf_ could not be calculated.

Secondary PK endpoints were time to maximum plasma concentration (T_max_), terminal rate constant (λ_z_), terminal half-life (t_1/2_), apparent volume of distribution during the terminal phase (Vz/F), apparent total plasma clearance (Cl/F), and percentage of AUC due to extrapolation from the last measured value to infinity (%AUC_extrap_).

Safety endpoints were the frequency of treatment-emergent adverse events, serious adverse events and adverse events of special interest, and the proportion of participants with clinically relevant changes in laboratory safety tests, vital signs (supine), or ECG (as triplicate) parameters. Adverse events of special interest were alanine transaminase (ALT) or aspartate transaminase (AST) > 3× the upper limit of normal (ULN) plus total bilirubin > 1.5×ULN, ALT or AST >8×ULN, or >3×ULN and symptomatic, uncorrected QT interval prolongation >500 msec, decline in hemoglobin ≥ 25% from baseline or an absolute value < 10 g/dL, clinically relevant decrease in neutrophil count, and platelet count ≥ 25% from baseline or an absolute value < 80 × 10^9^/L.

### Statistical analysis

For this pilot study with first administration of *Atoguanil* in humans, no formal statistical power calculation was done, and 16 participants were enrolled to have at least 14 participants complete. A previous assessment of *Malarone* PK in healthy adult participants indicated intersubject coefficient of variation (CV%) values of 20–23% for ATV and PG C_max_ and PG AUC_0-inf_, but > 30% for ATV AUC_0-inf_, suggesting that the proposed sample size would not allow for a formal bioequivalence assessment (Beerahee 1999). Thus, this study was conducted as an exploratory investigation to inform the design of a subsequent formal bioequivalence study.

The safety population included all randomized participants who received at least one dose of study treatment and was used for the safety analyses. The PK population was used for the PK analyses and included participants in the safety population with sufficient blood samples for calculation of at least one PK parameter.

PK parameters were estimated using non-compartmental analysis based on individual plasma concentration data, and the actual time of drug administration and blood sampling using Phoenix WinNonlin Version 8.3 (Certara, St Louis, USA). Samples below the LLOQ prior to the first quantifiable concentration were set to zero and those after the first quantifiable concentration were set to missing and omitted from the analysis. AUC was calculated using the linear/log trapezoidal method, applying the linear trapezoidal rule up to C_max_ and the log trapezoidal rule for the remainder of the curve. Other PK parameters were calculated according to standard equations and summarized using descriptive statistics.

F_rel_ at the doses tested was assessed as the ratio of the geometric means for exposure parameters expressed as a percentage, i.e., (*Atoguanil*/*Malarone)* × 100%. F_rel_ was calculated for ATV using for C_max_, AUC_0–t_, AUC_0–72h_, AUC_0–168h_, and AUC_0–inf_ and for PG and CG using C_max_ and AUC_0-inf_. Using the bioequivalence module within Phonenix WinNonlin, a linear mixed effect model was used to obtain the geometric means ratios, with the logarithm of the PK parameter as the response variable, the sequence, treatment, and period as fixed effects, and the subject within sequence as the random effect. Least square mean differences (*Atoguanil* − *Malarone*) were extracted from the model with 90% confidence intervals (CI). Mean ratios were reported with two-sided 90%CI after back transformation from the log-scale. For this pilot study, bioavailability was assumed to be similar if the 90%CI limits for the F_rel_ for each exposure parameter did not exceed 80% to 125%. A post hoc analysis was performed to determine dose-adjusted F_rel_ as the ratio of the geometric means for exposure parameters expressed as a percentage following formula: ((*Atoguanil*×1000)/(*Malarone*×500)) ×100%.

Adverse events were classified using the Medical Dictionary for Regulatory Activities (MedDRA, version 24.1). Adverse event severity was classified using a categorical grading system (mild/moderate/severe) based on a global clinical assessment by a trained research physician. All safety endpoints were analyzed using descriptive statistics using SAS Version 9.4 (SAS Institute, Cary, NC, USA).

## Results

### Participants

Of 38 individuals screened, 22 were excluded (5 reserve participants, 7 laboratory findings, 7 withdrew consent, 4 BMI/weight, 1 ECG, 1 medical history; including 3 re-screenings). All 16 enrolled participants completed the study and were included in the safety and PK populations. Mean (SD) age was 27.8 (4.9) years and 50% (8/16) were female; all females were of child-bearing potential (Table 2). The pre-specified threshold for a period 2 pre-dose ATV concentration > 5% of C_max_ was observed for one participant, who was excluded from the analysis of F_rel_ for both periods (modified PK population of 15 subjects). There were no other exclusions.

**Table 2:** Participant baseline demographic data (safety population)

Characteristic	*Atoguanil*–*Malarone* (*n* = 8)	*Malarone*–*Atoguanil* (*n* = 8)
Sex, *n* (%)		
Female	3 (37.5)	5 (62.5)
Male	5 (62.5)	3 (37.5)
Age, years		
Mean (SD)	26.1 (4.05)	29.5 (5.35)
Median (min, max)	25.5 (21, 33)	28.5 (24, 40)
Weight, kg		
Mean (SD)	69.7 (8.02)	59.7 (6.58)
Median (min, max)	70.3 (56.6, 79.8)	59.6 (49.5, 67.6)
Height, cm		
Mean (SD)	176.5 (7.46)	163.1 (8.49)
Median (min, max)	179.5 (166, 186)	162.5 (148, 178)
Body mass index kg/m^2^		
Mean (SD)	22.4 (1.86)	22.4 (1.55)
Median (min, max)	23.2 (19.4, 24.3)	22.7 (19.6, 24.8)
Ethnicity, *n* (%)		
Hispanic or Latino	8 (100)	8 (100)
Race, *n* (%)		
Asian	2 (25.0)	2 (25.0)
Black or African American	2 (25.0)	2 (25.0)
White	3 (37.5)	3 (37.5)
Other	1 (12.5)	1 (12.5)

*Max*, maximum; *min*, minimum; *SD*, standard deviation

### Pharmacokinetics

#### PK parameters

**Fig. 1:**
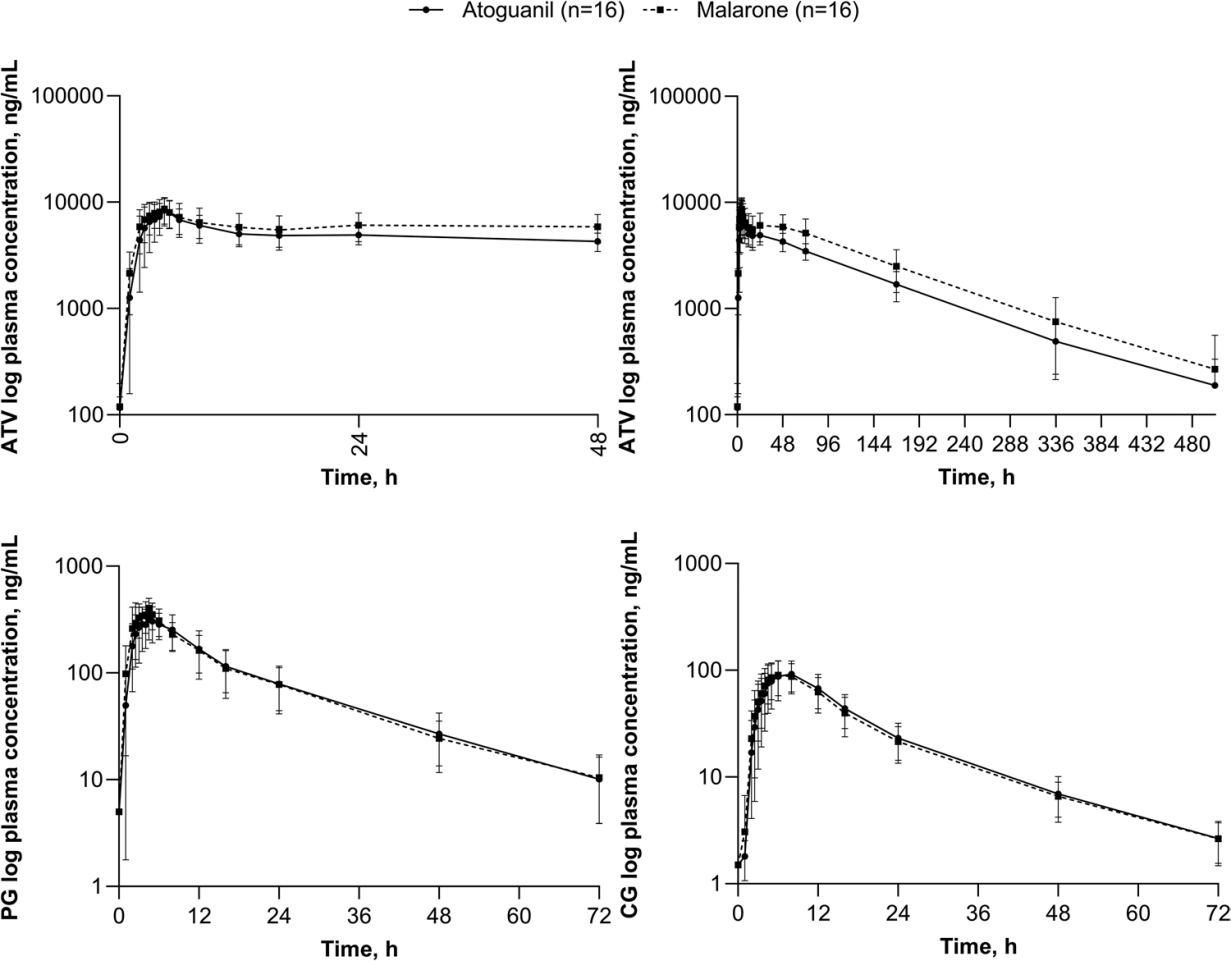
Mean (SD) plasma concentrations for ATV, PG, and CG over time following oral administration of *Atoguanil* (ATV 500 mg PG 348 mg) or *Malarone* (ATV 1000 mg plus PG hydrochloride 400 mg) in the presence of food (PK population, *n* = 16). ATV, atovaquone; PG, proguanil; CG, cycloguanil; SD, standard deviation

Following peak ATV plasma concentrations, plasma levels declined in a multiphasic manner and remained quantifiable up to 504 h post-dose in both treatment groups (Fig. 1). Measurable concentrations of PG and CG were present until at least 72 h post-dose with a T_lag_ of between 0.13 and 0.25 h with PG and 1.00 and 0.72 h for CG, reflecting the rapid metabolic conversion (Fig. 1). Median T_max_ of ATV after administration of *Atoguanil* and *Malarone* was 4.5 h for both products. PK parameters for ATV, PG, and CG following *Atoguanil* or *Malarone* are summarized in Table 3.

**Table 3:** Pharmacokinetic parameters for ATV, PG, and CG following administration of *Atoguanil* (ATV 500 mg PG 348 mg) or *Malarone* (ATV 1000 mg plus PG hydrochloride 400 mg) in the presence of food (PK population, *n* = 16)

Parameter	ATV		PG		CG	
	*Atoguanil*	*Malarone*	*Atoguanil*	*Malarone*	*Atoguanil*	*Malarone*
C_max_, ng/mL	8785 (2290)	9291 (2032)	376 (110)	444 (102)	95.4 (32.4)	94.7 (31.0)
AUC_0-t_, ng∙h/mL	775,142 (171,949)	1,085,759 (403,225)	5414 (2008)	5580 (1872)	1625 (550)	1586 (533)
AUC_0-72h_, ng∙h/mL	328,036 (57,217)	417,788 (119,298)	NC	NC	NC	NC
AUC_0-168h_, ng∙h/mL	566,232 (90,906)	770,758 (242,964)	NC	NC	NC	NC
AUC_0-inf_, ng∙h/mL	812,520 (184,824)	1,140,784 (453,803)	5720 (2090)	5884 (1953)	1693 (559)	1651 (543)
T_max_, h	4.50 (2.0, 8.0)	4.50 (2.5, 72.1)	4.50 (2.5, 8.0)	4.00 (2.0, 5.0)	8.00 (4.5, 8.1)	6.00 (4.0, 8.0)
T_lag_, h	0.06 (0.25)	0.06 (0.25)	0.25 (0.45)	0.13 (0.35)	1.00 (0.71)	0.72 (0.69)
λ_z_, 1/h	0.0080 (0.0024)	0.0081 (0.0023)	0.0483 (0.0095)	0.0469 (0.011)	0.0513 (0.011)	0.0473 (0.0072)
t_1/2_, h	94.6 (30.0)	93.2 (33.1)	14.9 (3.0)	15.5 (3.5)	14.1 (2.9)	15.0 (2.3)
Vz/F, mL	84,616 (18,752)	129,073 (45,469)	1,416,539 (395,769)	1,426,784 (495,489)	4,719,618 (2,164,154)	5,309,301 (2,796,912)
Cl/F, mL/h	657.4 (213.8)	1073.5 (645.1)	68,632 (24,275)	65,151 (20,365)	238,253 (120,014)	247,329 (129,630)
%AUC_extrap_, %	4.66 (4.18)	4.43 (4.11)	5.48 (2.12)	5.28 (3.04)	4.31 (1.66)	4.24 (1.90)

Data are mean (SD) except for T_max_ which is median (min, max)

*NC*, not calculated; *ATV*, atovaquone; *PG*, proguanil; *CG*, cycloguanil; *Cmax*, maximum observed plasma concentration; *AUC*, area under the plasma concentration–time curve; *AUC*_*0-t*_, AUC from time zero to last detectable plasma concentration; *AUC*_*0-72h*_, AUC from time zero to 72 h; *AUC*_*0-168h*_, AUC from time zero to 168 h; *AUC*_*0-inf*_, AUC from time zero to infinity; *T*_*max*_, time to maximum plasma concentration; *T*_*lag*_, time prior to the first measurable (non-zero) concentration; (*λ*_*z*_), terminal rate constant; *t*_*1/2*_, terminal half-life; *Vz/F*, apparent volume of distribution during the terminal phase; *Cl/F*, apparent total plasma clearance; *%AUC*_*extrap*_, percentage of AUC that is due to extrapolation from the last measured value to infinity

#### Relative bioavailability

Geometric means and intersubject variability (CV%) and the analysis of F_rel_ for PK exposure measures are shown in Table 4. At the doses tested, *Malarone* treatment gave rise to higher ATV exposure values when compared to *Atoguanil* with moderate CV%. ATV F_rel_ following *Atoguanil* was 93.6% (90%CI 83.6, 104.9) for C_max_ and 77.8% (90%CI 67.4, 89.8) for AUC_0-inf_. Thus, in this pilot study, at the doses tested F_rel_ between *Atoguanil* 500 mg and *Malarone* 1000 mg was similar for ATV C_max_, but not for AUC_0-inf_. When correcting for the ATV dose, based on AUC_0-inf_ the F_rel_ of *Atoguanil* was 156% compared to *Malarone*.

**Table 4:** Relative bioavailability of ATV, PG, and CG following administration of *Atoguanil* (ATV 500 mg PG 348 mg) or *Malarone* (ATV 1000 mg plus PG hydrochloride 400 mg) in the presence of food (modified PK population, *n* = 15)

Analyte	Exposure parameter	Geometric mean (CV%)		Geometric LSM^a^		F_rel_ (90% CI)^b^	Dose-adjusted F_rel_ (90%CI)^c^
		*Malarone*	*Atoguanil*	*Malarone*	*Atoguanil*		
ATV	C_max_, ng/mL	9090 (22.0)	8500 (30.4)	9191	8607	93.6 (83.6, 104.9)	187.3 (167.2, 209.8)
	AUC_0-t_, ng∙h/mL	998,000 (48.9)	752,000 (28.4)	997,432	772,976	77.5 (67.4, 89.2)	155.0 (134.8, 178.4)
	AUC_0-72h_, ng∙h/mL	401,000 (31.0)	323,000 (18.9)	394,901	327,685	83.0 (73.2, 94.1)	166.0 (146.4, 188.2)
	AUC_0-168h_, ng∙h/mL	730,000 (37.4)	558,000 (18.9)	724,249	570,198	78.7 (68.8, 90.1)	157.5 (137.6, 180.2)
	AUC_0-inf_, ng∙h/mL	1,050,000 (48.7)	789,000 (26.8)	1,034,239	804,620	77.8 (67.4, 89.8)	155.6 (134.8, 179.6)
PG^d^	C_max_, ng/mL	434 (22.5)	362 (28.8)	439	361	82.4 (75.8, 89.5)	164.5 (151.6, 179.0)
	AUC_0-inf_, ng∙h/mL	5600 (33.1)	5380 (37.2)	5686	5434	95.6 (92.1, 99.2)	191.1 (184.2, 198.4)
CG^d^	C_max_, ng/mL	88.6 (42.9)	89.7 (38.8)	88.4	89.1	100.8 (95.0, 107.0)	201.6 (190.0, 214.0)
	AUC_0-inf_, ng∙h/mL	1540 (42.9)	1590 (41.0)	1574	1620	102.9 (98.4, 107.7)	205.8 (196.8, 215.4)

^a^Geometric least square means (LSM) were computed for log transformed data

^b^Relative bioavailability (*Atoguanil*/*Malarone*)*100. *Atoguanil* and *Malarone* were considered to be similar if the 90%CIs were between 80 and 125% for the geometric mean ratio of the least square mean differences for each exposure parameter (highlighted with shading)

^c^Dose-adjusted relative bioavailability ((Atoguanil*1000)/(Malarone*500))*100

^d^AUC_0-72h_ geometric LSM could not be calculated for CG and PG

*CV%*, intersubject coefficient of variation; *ATV*, atovaquone; *PG*, proguanil; *CG*, cycloguanil; *C*_*max*_, maximum observed plasma concentration; *AUC*, area under the plasma concentration–time curve; *AUC*_*0-t*_, AUC from time zero to last detectable plasma concentration; *AUC*_*0-72h*_, AUC from time zero to 72 h; *AUC*_*0-168h*_, AUC from time zero to 168 hours; *AUC*_*0-inf*_, AUC from time zero to infinity; *F*_*rel*_, relative bioavailability

At the doses tested, PG C_max_ was lower following *Atoguanil* versus *Malarone* (82.4% 90%CI [75.8, 89.5]), though AUC_0-inf_ values were similar for the two formulations (Table 4). PK exposure parameters for CG were similar for *Atoguanil* and *Malarone* (Table 4).

### Safety

Following *Atoguanil*, four adverse events (3 COVID-19 infection, 1 soft tissue infection) occurred in 25.0% (4/16) of participants. Following *Malarone*, five adverse events (1 diarrhea, 3 headache, 1 contact dermatitis) occurred in 25.0% (4/16) of participants. All adverse events were of mild severity, two cases of headache were treated symptomatically with fluid/analgesics, and all resolved by period end. None of the adverse events was considered related to study medication. There were no serious adverse events or deaths. There were no adverse events of special interest reported.

There were no clinically significant changes in biochemistry, hematology, or coagulation parameters. There were no clinically relevant changes in ECG parameters. One participant receiving *Malarone* had a single reading of a transient prolongation of Fridericia-corrected QT interval of 454 msec (53 msec increase from baseline) at 6 h post-dose on Day 1, which was not observed in nine repeated readings and not considered clinically relevant. No clinically relevant observations were made for physical examination or vital signs.

## Discussion

This open-label, single-dose, standard non-replicated randomized 2-period, 2-treatment balanced crossover pilot study evaluated the oral PK profile and F_rel_ of a novel formulation of ATV-PG (*Atoguanil*) relative to ATV-PG_HCl_ (*Malarone*) in healthy adults in the fed state. In vitro dissolution data of *Malarone* vs *Atoguanil* tablets in two discriminating dissolution media showed dissolution rates for *Atoguanil* were about 2 to 2.5 times that of *Malarone* (Medicines for Malaria Venture, data on file). Also, a study in rabbits showed similar bioavailability of 50 mg/kg *Atoguanil* to 100 mg/kg *Malarone* with the ratio for ATV AUC_0-inf_ being 99.6% (95%CI 96.4, 103.0) (personal communication, Byju Thankachen). Thus, the maximum theoretical systemic exposure to ATV from *Atoguanil* was expected to be approximately twofold higher than for the *Malarone*. In this study, the proposed *Atoguanil* ATV dose was 500 mg, anticipating similar ATV exposures after oral administration of the *Malarone* ATV dose of 1000 mg.

PK parameters for ATV, PG, and CG following *Malarone* were consistent with previous reports in healthy participants in the fed state (Gillotin et al. 1999). At the doses tested, F_rel_ was similar between *Atoguanil* and *Malarone* for C_max_ (93.6% [90%CI 83.6, 104.9]), but not for AUC_0-inf_ (77.8% [90%CI 67.4, 89.8]). The PG dose in *Atoguanil* was similar to that in *Malarone*, and PG and CG exposures were expected to be similar. This was the case except for the PG C_max_, with an F_rel_ of 82.4% (95%CI 75.8, 89.5).

The preferred study design for evaluating the F_rel_ is the cross-over study design. As ATV has a half-life of up to 116 h, a minimum wash-out interval of 24 days between the administration of two single doses was used. However, one participant had an ATV concentration > 5% of C_max_ in the second period and data from both periods were excluded from the F_rel_ analysis (modified PK data set).

The individual ATV concentration–time profiles following *Malarone* showed some transient plateau and secondary peaks after 24 h, observed nominally around the time the participants would be administered food. This observation may account for the higher ATV AUC following administration of *Malarone* versus *Atoguanil*. However, as the ATV dose in *Atoguanil* was 500 mg versus 1000 mg in *Malarone*, the dose-adjusted F_rel_ for ATV was improved by 156% with *Atoguanil* based on AUC_0-inf_. The ATV AUC_0-inf_ following administration of *Atoguanil* also showed a reduced variability compared to *Malarone*, with CV% values of 26.8% and 48.7%, respectively.

Only nine treatment-emergent adverse events were reported, with no apparent differences observed between the treatment groups. All adverse events were mild and not considered treatment related. No clinically relevant abnormalities were observed for clinical laboratory tests, hematology, vital signs, or ECG. Overall, the *Atoguanil* safety and tolerability profile was acceptable and comparable to that of *Malarone*.

Key limitations were that this is a pilot study with moderate intersubject variability observed for exposure parameters. The ATV AUC_0-inf_ CV% observed in our study was largely above 30%, confirming previous data in healthy participants (Beerahee 1999). This suggests that a replicate design in which the reference product is given more than once, or even a two-stage design, would probably be required for a formal bioequivalence study (United States Food and Drug Administration 2022). According to the European Medicines Agency guidelines, widening the conventional 20% acceptance range for bioequivalence based on high variability is possible for C_max_ but not AUC, and only if a replicate design is conducted (Committee for Medicinal Products for Human Use 2010). In contrast, the US Food and Drug Administration allows widening of the bioequivalence acceptance criteria for both C_max_ and AUC, as well as applying slightly different approaches to estimate within-subject variation for the reference product above 25% (Endrenyi and Tothfalusi 2019). Importantly, this study used a high-fat, high calorific meal. As there was moderate intersubject variability in ATV exposures following *Malarone* and transitory secondary peaks in ATV concentrations around the time the participants were administered food, the possibility that there would be similar F_rel_ between *Atoguanil* and *Malarone* following a low-fat meal or a milky drink as per normal *Malarone* dosing recommendations cannot be discounted, but this was not evaluated in this study. In terms of relevance to the target population, perennial malaria chemoprevention is recommended for children up to 24 months of age, and SMC is recommended for children under 5 years old. However, the safety and effectiveness of *Malarone* has not been confirmed in children weighing less than 11 kg. This study was necessarily conducted in healthy adults for ethical reasons.

## Conclusions

In this pilot study, the ATV F_rel_ following *Atoguanil* (ATV 500 mg PG 348 mg) or *Malarone* (ATV 1000 mg plus PG hydrochloride 400 mg) at the doses tested was not similar when both treatments were administered with high-fat/high caloric meal, based on AUC_0-inf_. However, given that *Malarone* contains twice the ATV dose of *Atoguanil,* the dose-adjusted F_rel_ of *Atoguanil* was 156% compared to *Malarone*. Both drugs were well tolerated with no safety concerns. However, the feasibility of halving the ATV dose required with the *Atoguanil* formulation compared with *Malarone* was not established.

## Data Availability

De-identified participant data are available on reasonable request and with completion of a signed data access agreement from (https://www.mmv.org/about-us/contact-us) referencing this publication. Data will be available for at least five years from publication of this study.
